# A case report of Cronkhite–Canada syndrome first encounterd at a hospital in northern Vietnam

**DOI:** 10.1097/MS9.0000000000001334

**Published:** 2023-09-20

**Authors:** Long Cong Nguyen, Thuy Thi Pham, Tung Thanh Nguyen, Nam Hoai Nguyen, Tuan Van Kieu, Giang Anh Do, Ha Thi-Ngoc Doan, Chuong Van Tran, Nhung Thi Vu

**Affiliations:** a Gastroenterology and Hepatology Center; b Pathology and Cytology Center, Bach Mai Hospital; c Department of Internal Gastroenterology, University of Medicine and Pharmacy, Hanoi National University, Hanoi, Vietnam

**Keywords:** case report, cronkhite–canada syndrome, hyperpigmentation, onychodystrophy, polyposis

## Abstract

**Introduction and importance:**

Cronkhite–Canada syndrome (CCS) is an extremely rare non-inherited syndrome first described in 1955 with only about 500 more cases reported so far. Since the aetiology of the disease remains unknown, there were no specific treatments in consensus. In many countries, CCS is a completely new condition that may confuse physicians at first encounter. Lessons should be learned from these cases by gastrointestinal specialists to be aware of this condition in any circumstances.

**Case presentation:**

The authors reported a case study of a 45-year-old Vietnamese male with CCS diagnosis, which encountered at our centre for the first time.

**Clinical discussion:**

The definitive diagnosis was provided by combining clinical characteristics, and endoscopic and histopathologic features, after excluding other causes of gastrointestinal polyposis. The patient responds to corticosteroids, proton pump inhibitors, and nutritional support right after treatment. After 1 year of treatment, his symptoms ameliorated completely although colon polyps insignificantly reduced.

**Conclusion:**

Gastroenterologists should always be aware of patients with CCS with the following symptoms: gastrointestinal hamartomatous polyps, diarrhoea, and the dermatologic triad of alopecia, hyperpigmentation, and onychodystrophy.

## Introduction

Highlights

- Cronkhite–Canada syndrome is an extremely rare non-inherited syndrome.
- In many countries, Cronkhite–Canada syndrome is a completely new condition that may confuse physicians at first encounter.
- The definitive diagnosis was provided by combining clinical characteristics, endoscopic and histopathologic features
- The patient responds to corticosteroids and ameliorated completely after 1 year of treatment.

Cronkhite–Canada syndrome (CCS) is a rare non-hereditary disease. The disease was first reported in 1955 in the New England Journal of Medicine by Leonard Wolsey Cronkhite and Wilma Jeanne Canada^1^. Since then, only about 500 more cases have been described in the medical literature in which about 75% of cases were reported in Japan^2,3^. The disease is characterized by gastrointestinal symptoms including diarrhoea, abdominal pain, dysgeusia, and weight loss. Later, the dermatologic triad of hyperpigmentation, alopecia, and dystrophic nails often occurs^4^. Endoscopy examination often found multiple diffuse gastrointestinal sessile polyposes except in the oesophagus. Gastric and colon histology was benign Juvenile polyposis—like hamartomatous polyps, infiltration of inflammatory cells including eosinophils^2^.

Since its manifestation happens in multiple organs unspontaneously, it sometimes challenges clinicians to provide a definitive diagnosis, especially in medical facilities that encounter the disease for the first time. Here we would like to describe a case report of Cronkhite Canada first presented at our centre, the largest gastroenterology centre in Northern Vietnam. The patient was diagnosed at our centre when the symptoms became clearer, after 4 months of clinical manifestation and then treatment at another national hospital. However, since it was the initial case, our centre still needed several endoscopy and pathology performances to provide the final definitive diagnosis.

## Case report

The patient provided informed consent. Ethics approval is not required for case reports at our institution. The case report was written according to the Care checklist^5^.

**Figure 1:**
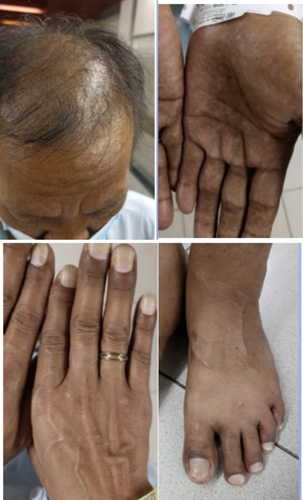
Hair and eyebrow loss; atrophy of fingernail, toenails, and palmar hyperpigmentation.

A 45-year-old male visited our hospital in February 2022 with primary gastrointestinal complaints of diarrhoea about 10 times a day for 4 months, sometimes with blood. The patient felt fatigue, dull epigastric pain, appetite loss, and lost 11 kg in 2.5 months. Later, he noticed several ectodermal changes. Hyperpigmentation of the skin appeared in fingers, tongues, lips, and face (Fig. 1). He gradually lost his hair and eyebrows. He also denied any fever, chills, nausea, vomiting, or dysphagia. He had no history of food allergy, no alcohol drinking, and no smoking. In his family, no one had polyposis in the stomach and colon.

Two months before admission to our hospital, the patient was examined at another hospital with the diagnosis of ulcerative colitis, gastroesophageal reflux disease, and toxocariasis infection. He was treated with mesalazine 1.5g bid in 2 months, proton pump inhibitor in 1 month, and albendazole 400 mg bid in 7 days but his symptoms did not improve.

When admitted to our department, a physical examination showed that the patient was malnourished with a BMI of 17 kg/m^2^, had jaundice, and leg oedema.

The patient’s blood test results showed hypoalbuminemia, serum IgG4 was within normal limits, no heavy metal contamination, and negative with autoimmune marker tests (Table 1).

**Table 1:** Results of patient’s blood tests at admission.

Laboratory test	Results	Normal range
Blood cell count (cells/l)	7.45×10^9^	4–10×10^9^
Platelet (cells/l)	285×10^9^	100–400×10^9^
Haemoglobin (g/l)	131	135–175
C-reactive protein (mg/l)	Normal	<10
Albumin (g/l)	20.4	35–52
Total protein (g/l)	43.3	66–83
Serum iron (mcmol/l)	24.2	8.1–28.6
Serum ferritin (mcg/l)	345	23.9–336.2
Cortisol (nmol/l)	357.3	185–624
ACTH (pg/ml)	24.09	7.2–63.3
Antinuclear antibody (ANA)	Negative	Negative
Antimitochondrial antibody (AMA)	Negative	Negative
Smooth-muscle antibody (SMA)	Negative	Negative
Antineutrophil cytoplasmic antibody (ANCA)	Negative	Negative
Blood Cu	Normal	Normal
Urine Cu	Normal	Normal
Blood Pb	Normal	Normal
Urine Pb	Normal	Normal
Blood Asen	Normal	Normal
Urine Asen	Normal	Normal
IgG4	Normal	Normal
Vitamin B12	Normal	Normal

ACTH, Adrenocorticotropic hormone.

**Figure 2:**
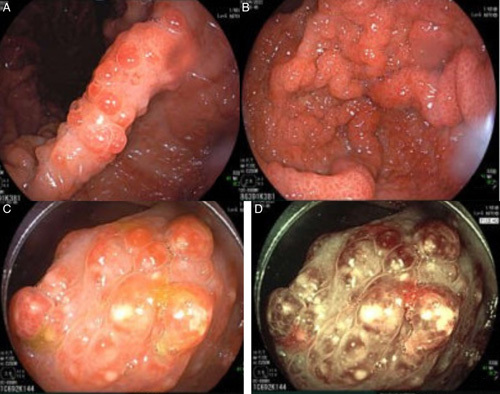
(A, B) The first esophagogastroduodenoscopy revealed inflammation of the entire gastric mucosa, concomitant without pedunculate, wide polyposis, size 0.5–1.0 cm, not excluding lymphoma or Peutz-Jeghers syndrome, (C, D) The first colonoscopy revealed inflammation of the entire colon mucosa, concomitant without pedunculate polyposis, size 0.5–1.0 cm, not excluding lymphoma or Peutz-Jeghers syndrome.

Gastric endoscopy showed erythema and oedema in the entire mucosal surface. Noticeably, there we many small polypoid nodules spread over both gastric and colon surfaces, including the terminal ileum (Fig. 2). No lesions were noted in the oesophageal mucosa.

Biopsy specimens from the gastric antrum mucosa show a hamartomatous polyp with cystically dilated glands and crypts associated with an oedematous lamina propria containing small blood vessel proliferation, inflammatory cell infiltration including mononuclear cells, and eosinophils.

**Figure 3:**
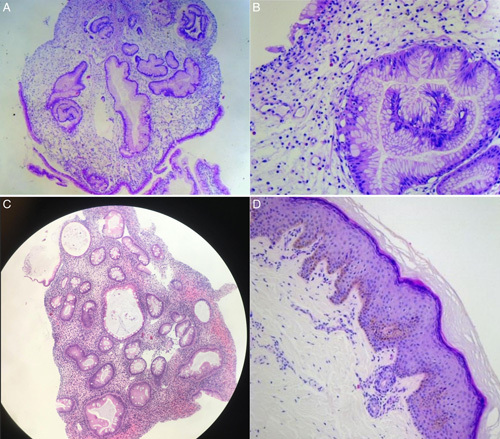
Biopsy specimen from gastric antrum mucosa displayed a hamartomatous polyp with an expanded lamina propria and regional glandular epithelial hyperplasia. Glands show excessive branching and dilation [(A) hematoxylin and eosin (H&E) × 50] mucosal chronic inflammation, oedematous stroma with inflammatory cell infiltration, and small blood vessel proliferation [(B) H&E ×200]. Colon histopathology displayed mucosal chronic inflammation and colon ulcer. Epitheliums were mildly slick. Epithelial cells had small, polar nuclei, stromal infiltrates with many lymphocytes, eosinophils, some neutrophils, and plasma cells. No granulitis. No malignant cells [(C) H&E ×200]. Skin biopsy shows hyperpigmentation. Elongation of the rete ridges and increase in number of melanocytes in the basal layer. Mild epidermal acanthosis, increased numbers of uniformly dispersed single melanocytes without atypia in the basal layer, and variable basal hyperpigmentation [(D) H&E ×200].

The patient also underwent abdominal ultrasound, abdominal computed tomography, and skin biopsy (Fig. 3D). The results showed no evidence of lymphoma, inflammatory bowel disease, parasitic infection, Wilson disease, hemochromatosis disease, Addison’s disease, or Menetrier disease.

Combining the histopathological results with typical clinical signs, we considered the patient to have Crohnkhite Canada syndrome. Other polyposis syndromes consisting of Peutz-Jeghers syndrome, juvenile polyposis, familial adenomatous polyposis, hyperplastic polyposis, and Cowden disease were also considered and finally excluded based on the age of onset, inherited polyposis, characteristic of polyps, the number of polyps, the size of polyps, the common location of polyps and clinical features.

The patient was treated with corticosteroids at a dose of 1 mg per kg body weight daily, proton pump inhibitors (PPI) (esomeprazole 40 mg once a day), and nutrition support.

After 1 month of treatment, his gastrointestinal symptoms ameliorated gradually. His diarrhoea improved markedly within a short period five times a day. He regained his appetite gradually and his weight gained ~5 kg. However, his ectodermal disorders have not recovered yet. Also, nail and toenail loss were observed.

Laboratory tests showed normalisation of serum albumin levels returned. The second esophagogastroduodenoscopy and colonoscopy were also performed to evaluate the gastrointestinal tract again. The lesions in gastric and colon did not change much compared to pre-treatment. However, based on the improvement in clinical symptoms, we discharged the patient within one month of treatment.

Outpatient management was maintanined for six more months with corticosteroids 1 mg/kg/day (Medrol 16 mg three times a day), PPI (esomeprazole 40 mg once a day), nutritional support, and capsules to modulate intestinal flora. Corticosteroids then decreased to twice a day in 4 months.

**Figure 4:**
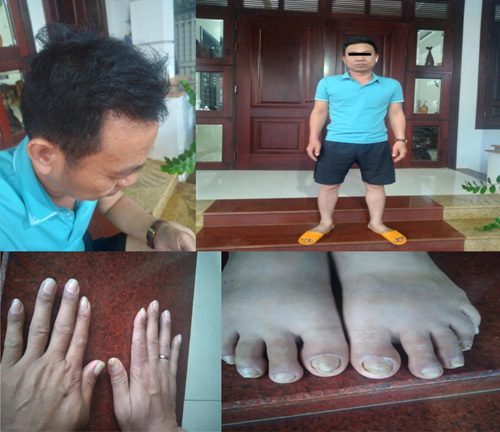
Patient after 1 year of treatment. His hair, nails, and eyebrows grew back, and pigmentations disappeared.

**Figure 5:**
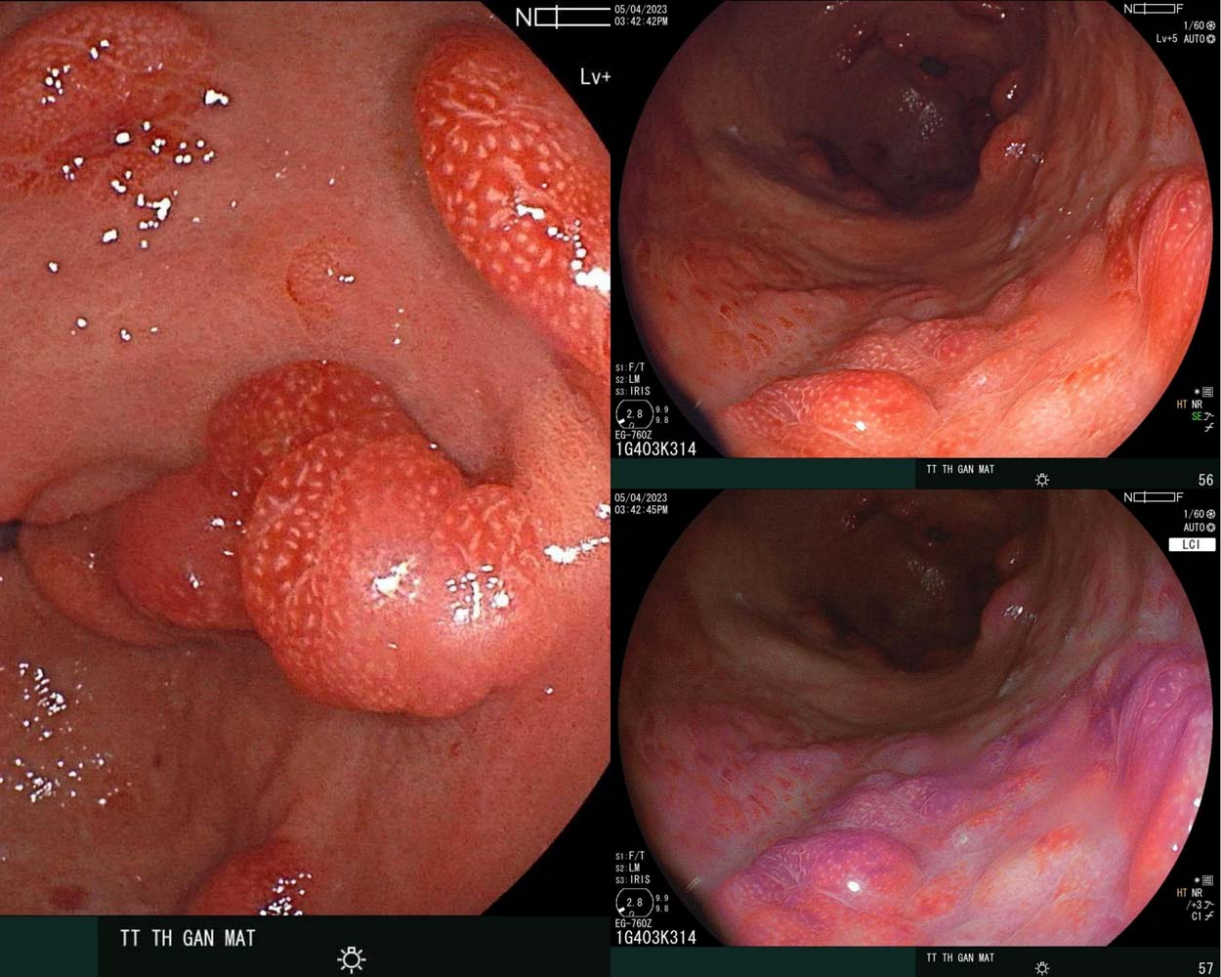
Gastric endoscopy after 1 year of treatment.

**Figure 6:**
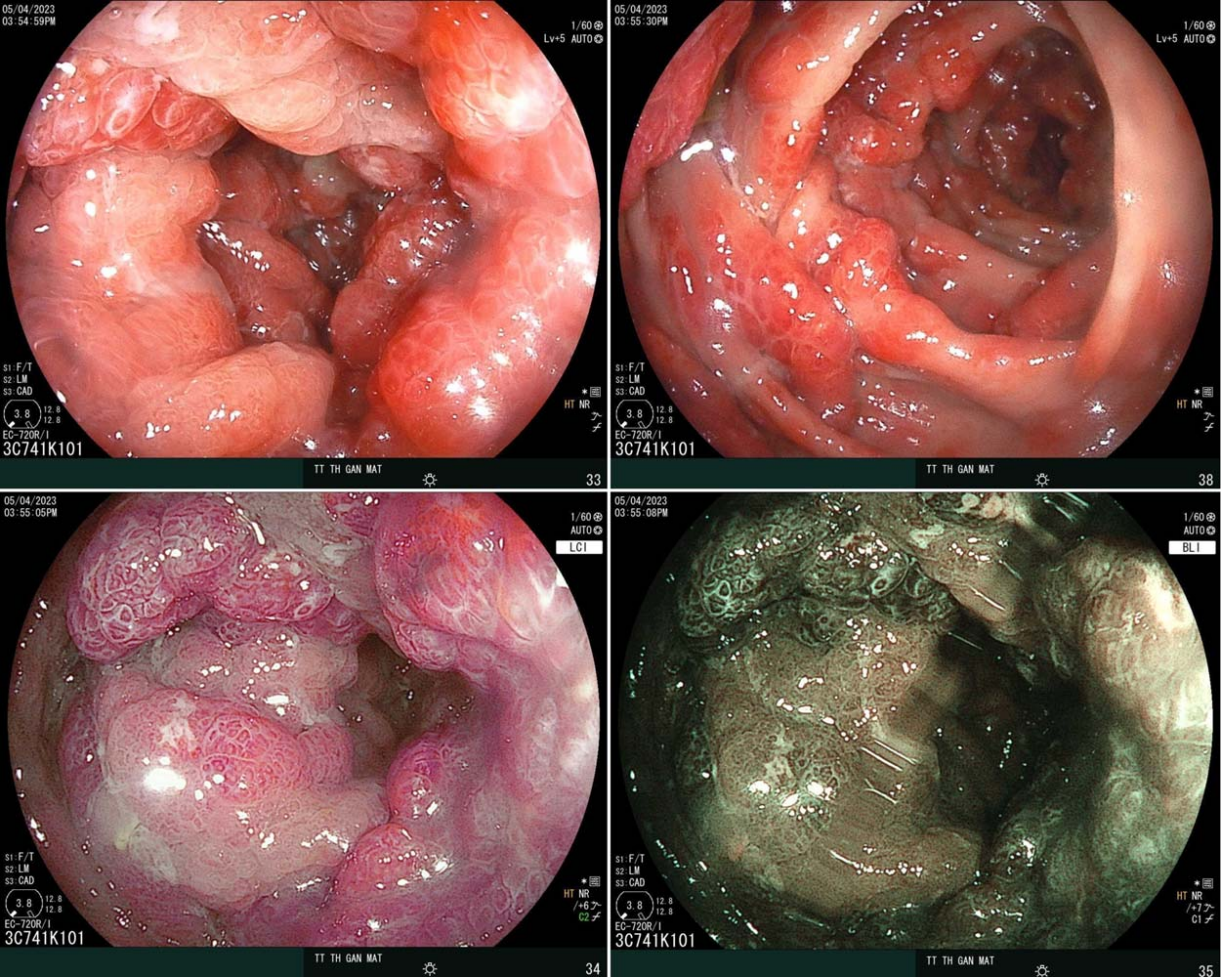
Colon endoscopy after 1 year of treatment.

After 1 year of treatment with corticosteroid, PPI, and nutritional support (human albumin), his weight gained from 49 to 68 kg. He regained his appetite. The diarrhoea disappeared and is now maintained only once a day. Palmar, feet, lip, and lower limb pigmentations disappeared. His hair and eyebrows grew back. His toe and toenails also recovered (Fig. 4). In gastrointestinal endoscopy, polyps are relatively reduced in the stomach but remain abundantly in the colon (Figs. 5,6).

## Discussion

CCS is a rare multiple gastrointestinal polyps and its aetiology and pathogenesis are currently unclear^6^. Gastrointestinal symptoms are the main manifestations, including abdominal pain, diarrhoea, and dysgeusia. Diarrhoea is the most common initial symptom, which may develop into substantially watery diarrhoea, followed by symptoms of malabsorption, including weakness, anaemia, weight loss, and oedema due to hypoalbuminemia, especially in the eyelids and extremities^7^. The dermatologic triad of hyperpigmentation, alopecia, and dystrophic nails often appear later^8^.

Gastric and colon endoscopy reveal sessile polyps in the stomach, small bowel, and colorectum but not in the oesophagus^7^. Polyps are non-neoplastic hamartomas but about 15% of CCS developed cancerous polyps^9^. Histopathologic results of polyps revealed cystic dilation of the mucosal gland with inflammatory infiltration including eosinophils, massive submucosal oedema mostly located in the lamina propria, hyperplasia of the foveolar epithelium, focal hyperplastic, all of these features were not typical^10^.

In this case, the patient is male, 45 years old. He was diagnosed and treated in another hospital but his symptoms did not improve. After that, he was admitted to our hospital with a typical dermatologic triad including hair and eyebrow loss, nail and toenails atrophy, and skin hyperpigmentation in palms and feet which exhibited gastrointestinal symptoms including diarrhoea, nausea, fatigue, and appetite loss. Possibly, at the time of visiting our centre, the disease had progressed to the late stage, with more typical symptoms. Therefore, it is easier to suspect the patient with CCS.

This was the first case diagnosed with CCS at our hospital. Noticeably, gastric and colon endoscopy and histology reveal typical features of CCS for definitive diagnosis, with the infiltration of inflammatory cells including eosinophils and images of potential multiple polyposis. As the first time encountering this condition, differential diagnosis was carefully considered between CCS and several polyposis syndromes, including familial adenomatous polyposis, Peutz-Jeghers syndrome, Cowden disease, and juvenile polyposis. The differential diagnosis was made based on the age of onset, inherited polyposis, characteristics of polyps, the number of polyps, the size of polyps, the common location of polyps, and clinical features^8,11^. Furthermore, protein-losing enteropathies such as chronic inflammatory bowel diseases, Whipple’s disease, or intestinal lymphangiectasia have also been ruled out^12^. Combined with the typical clinical symptoms, we finally confirmed the diagnosis of CCS.

Although the incidence of CCS is low, it is associated with high mortality as a result of malnutrition, severe electrolyte imbalance, gastrointestinal bleeding, anaemia, infection, and shock; the 5-year mortality may be as high as 55%^7^. One of the reasons is the risk of developing gastric and colon cancer of up to 15%^13^, which would be early screened by annual endoscopic surveillance. In the case of gastric cancer, even if dysplastic lesions appeared, total gastrectomy is usually indicated. For colon cancer, depending on the location of the lesions, treatment methods would be subtotal or total proctocolectomy^14^. Ward and Wolfsen recommend systematically resecting all polyps greater than 1.0 cm to prevent the development of colorectal carcinoma^15,16^. Fortunately, the patient did not have any cancerous polyposis in histology.

There is no consensus on the treatment of CCS. In general, the patients received immunosuppressive therapy with corticosteroids or long-term azathioprine therapy, nutritional and micronutrient supplements, in addition to endoscopic monitoring and resection of the remaining large polyps to prevent cancer^6,9,17^. In this case, we treated the patient with a corticosteroid, nutritional supplementation, or a nutritionally balanced liquid diet, antibiotics, PPI, and capsules to modulate intestinal flora control symptoms and provide support. After 1 month, his symptoms were relieved, and the patient’s appetite gradually improved, resulting in a total recovery of clinical manifestation 1 year after treatment. In gastrointestinal endoscopy 1 year after treatment, polyps are significantly reduced in the stomach but remain in the colon. In a Japanese study of gastric polyps, it took on average 248 days for gastric polyps and 238 days for colon polyps to decrease in size and number^3^.

Conventionally, regardless of treatment, the prognosis of CCS patients with a 5-year mortality is as low as 55%, in which 5–10% of cases observed regressions^18^. However, a recent report showed a remarkable improvement in 5-year survival of nearly 90%, with glucocorticoid treatment^19^, bringing more expectations to patients with this condition.

## Conclusion

Patients with gastrointestinal hamartomatous polyps, diarrhoea, and the dermatologic triad of alopecia, hyperpigmentation, and onychodystrophy should be considered for the diagnosis of CCS. CSS diagnosis should be provided by exclusion of other polyposis. The mainstay treatment of corticosteroids or long-term azathioprine therapy would result in the remission of the disease.

## Ethical approval

Ethics approval is not required for case reports at our institution.

## Consent

Written informed consent was obtained from the patient for the publication of this case report and accompanying images. A copy of the written consent is available for review by the Editor-in-Chief of this journal on request.

## Source of funding

None.

## Author contribution

L.C.N.: conceptualization, supervision, validation, visualization, writing—original draft, writing—review and editing. T.T.P., T.T.T.: conceptualization, software, validation. N.H.N., T.V.K.: visualization, writing—original draft, writing—review and editing. G.A.D., H.T.N.D.: formal analysis, investigation, methodology, software. C.V.T.: data curation, formal analysis, investigation, writing—review and editing. N.V.T.: methodology, writing—original draft.

## Conflicts of interest disclosure

The authors declare that they have no conflict of interest.

## Research registration unique identifying number (UIN)

1. Registry used: www.researchregistry.com.
2. Unique Identifying number or registration ID: researchregistry9086.
3. Hyperlink to your specific registration (must be publicly accessible and will be checked): Browse the Registry- Research Registry.

## Guarantor

Nguyen Cong Long.

## Provenance and peer review

Not commissioned, externally peer-reviewed.

## Data availability

Data are available upon reasonable request.
